# Regulatory T Cells and Human Myeloid Dendritic Cells Promote Tolerance via Programmed Death Ligand-1

**DOI:** 10.1371/journal.pbio.1000302

**Published:** 2010-02-02

**Authors:** Shoba Amarnath, Carliann M. Costanzo, Jacopo Mariotti, Jessica L. Ullman, William G. Telford, Veena Kapoor, James L. Riley, Bruce L. Levine, Carl H. June, Timothy Fong, Noel L. Warner, Daniel H. Fowler

**Affiliations:** 1 Experimental Transplantation and Immunology Branch, National Cancer Institute, National Institutes of Health, Bethesda, Maryland, United States of America; 2 University of Pennsylvania, Abramson Family Cancer Research Institute, Philadelphia, Pennsylvania, United States of America; 3 BD BioSciences, San Jose, California, United States of America; National Jewish Medical and Research Center/Howard Hughes Medical Institute, United States of America

## Abstract

Immunotherapy using regulatory T cells (Treg) has been proposed, yet cellular and molecular mechanisms of human Tregs remain incompletely characterized. Here, we demonstrate that human Tregs promote the generation of myeloid dendritic cells (DC) with reduced capacity to stimulate effector T cell responses. In a model of xenogeneic graft-versus-host disease (GVHD), allogeneic human DC conditioned with Tregs suppressed human T cell activation and completely abrogated posttransplant lethality. Tregs induced programmed death ligand-1 (PD-L1) expression on Treg-conditioned DC; subsequently, Treg-conditioned DC induced PD-L1 expression in vivo on effector T cells. PD-L1 blockade reversed Treg-conditioned DC function in vitro and in vivo, thereby demonstrating that human Tregs can promote immune suppression via DC modulation through PD-L1 up-regulation. This identification of a human Treg downstream cellular effector (DC) and molecular mechanism (PD-L1) will facilitate the rational design of clinical trials to modulate alloreactivity.

## Abstract 2

Human regulatory T cells inhibit graft-versus-host disease that can occur after tissue transplantation, in part through expression of programmed death ligand 1 and modulation of antigen-presenting cells.

## Author Summary

Graft-versus-host disease (GVHD) is the most serious complication of bone marrow transplants between individuals (so-called allogenic transplants). The class of suppressor immune cells called regulatory T cells (Tregs) inhibit GVHD by dampening the effects of donor immune cells in the grafted tissue. The cellular and molecular mechanisms involved in this process have not been fully characterized, particularly for human cells. In this study, we report that human Tregs, which we generated from precursor cells ex vivo, express high levels of a cell surface protein called PD-L1 (programmed death ligand-1) that is known to mediate immune suppression. Coculture of these Tregs with allogeneic antigen-presenting cells (APCs), which are known to initiate GVHD, increased, in turn, the amount of PD-L1 on the APCs. The Treg-conditioned APCs were then less able than unconditioned APCs to provoke GVHD in a mouse model of the condition, preventing the death of the animals after transplantation. We found that an antibody against PD-L1 blocked the immunosuppressive effects of Tregs or Treg-conditioned APCs, indicating that this protein is an important part of the molecular mechanism. These findings are potentially important for attempts to modulate immune responses in disease by transplanting T cells into patients.

## Introduction

Regulatory T cells (Tregs) promote immune tolerance to self-antigens and alloantigens (reviewed in [1]). Genetic deficiency of Tregs mediated by lack of Foxp3 transcription factor yields autoimmunity in mice [2] and humans [3]. Numerical or functional deficiency of Tregs in murine models exacerbates autoimmune disease [4],[5], predisposes to solid organ and hematopoietic stem cell graft rejection [6],[7], and associates with acute and chronic graft-versus-host disease (GVHD) [8]–[10]. Importantly, clinical studies have demonstrated Treg defects in humans with autoimmune disease [11],[12] and GVHD [13]–[15]. Given this background, a rationale has been outlined to evaluate adoptive cell therapy using ex vivo–expanded Tregs as an approach to treat autoimmune [16] or alloimmune [17] conditions. Negative selection against the IL-7 receptor alpha chain (CD127) enriches for human Tregs [18] and thereby may represent a useful tool for such cell therapy efforts; however, there are currently no reports pertaining to the regulatory function of cells expanded from CD127-depleted human T cells. Given this information, our experiments focused on human Tregs generated ex vivo by enrichment for CD127-depleted CD4^+^ T cells and by culture in conditions demonstrated to promote Treg expansion, including CD28 costimulation IL-2, TGF-β [19], and rapamycin [20].

A more comprehensive understanding of cellular and molecular mechanisms of adoptively transferred Treg products would facilitate the rational design of clinical trials evaluating Tregs. Such an understanding may be difficult to ascertain given the varieties of Tregs [21] and numerous molecular mechanisms operational in murine Treg cells, including: CTLA-4 [22], TGF-β [23], PD-L1 [24], GITR [25], or IL-10 [9]. The cellular mechanism of Tregs also is complex and varied depending on the particular experimental model; importantly, recent evidence indicates that murine Tregs inhibit responder T cells indirectly via modulation of dendritic cells (DC) [26],[27].

Identification of cellular and molecular mechanisms of human Tregs, in particular ex vivo–generated Tregs, has been relatively elusive. For example, ex vivo–generated human Tregs suppressed an allogeneic mixed lymphocyte reaction (allo-MLR) by an undefined mechanism that operated independent of IL-10 or TGF-β [28]. Indeed, the role of antigen-presenting-cell (APC) modulation as a human Treg mechanism has been somewhat neglected in part because published studies have typically utilized APC-free suppressor assays. Nonetheless, one recent study determined that freshly isolated Tregs inhibited myeloid DC inflammatory cytokine secretion and costimulatory molecule expression; such Treg-conditioned DC had reduced capacity to stimulate alloreactivity in vitro [29]. In light of this relative paucity of information relating to the mechanism of ex vivo–generated human Tregs, our primary objective was to elucidate the cellular and molecular pathways associated with human Treg cell suppressor function. Because of our focus on allogeneic hematopoietic stem cell transplantation (HSCT), the role of Tregs in GVHD protection, and the role of host APC for GVHD induction [30], we elected to study human Tregs in vitro using an allo-MLR driven by a defined population of myeloid DC and in vivo using a xenogeneic GVHD (x-GVHD) model similar to that previously utilized to study human Tregs [31].

## Results

### CD127 Negative Selection and Expansion Yields a Human Treg Phenotype

Total CD4^+^ and CD4^+^CD127^−^ T cells were costimulated and expanded in medium containing IL-2, TGF-β1, and rapamycin to generate control bulk “CD4” and “Treg” populations that were directly compared in each experiment. Expanded T cells maintained their CD127^−^ status, were comparable in terms of expansion (Figure S1A), coexpression of CD62L with CCR7 (Figure S1B (i)) and Foxp3 expression (Figure S1B (ii)). Because Foxp3 is expressed in human Tregs and transiently expressed in human effector T cells [32], we reasoned that bulk CD4 cell Foxp3 content may represent a marker of effector differentiation. To address this, we compared ex vivo–expanded T cells for simultaneous expression of Foxp3 and effector cytokines, including IL-2 (Foxp3^+^IL-2^+^ events) and IFN-γ (Foxp3^+^IFN-γ^+^ events). Indeed, relative to Tregs, control CD4 cells had increased coexpression of Foxp3 with IL-2 (Figure S1C (i)) and Foxp3 with IFN-γ (Figure S1C (ii)). Furthermore, relative to control CD4 cells, expanded Tregs mediated increased suppression of CD4^+^ and CD8^+^ T cell alloreactivity (Figure S1D (i) and S1D (ii)); suppression was observed at a Treg cell to responder T cell ratio of 1∶20 that approximates the physiologic ratio (see dose-response curve, Figure S1E).

### Treg Cells Modulate the Programmed Death-1 (PD-1) Pathway

**Figure 1:**
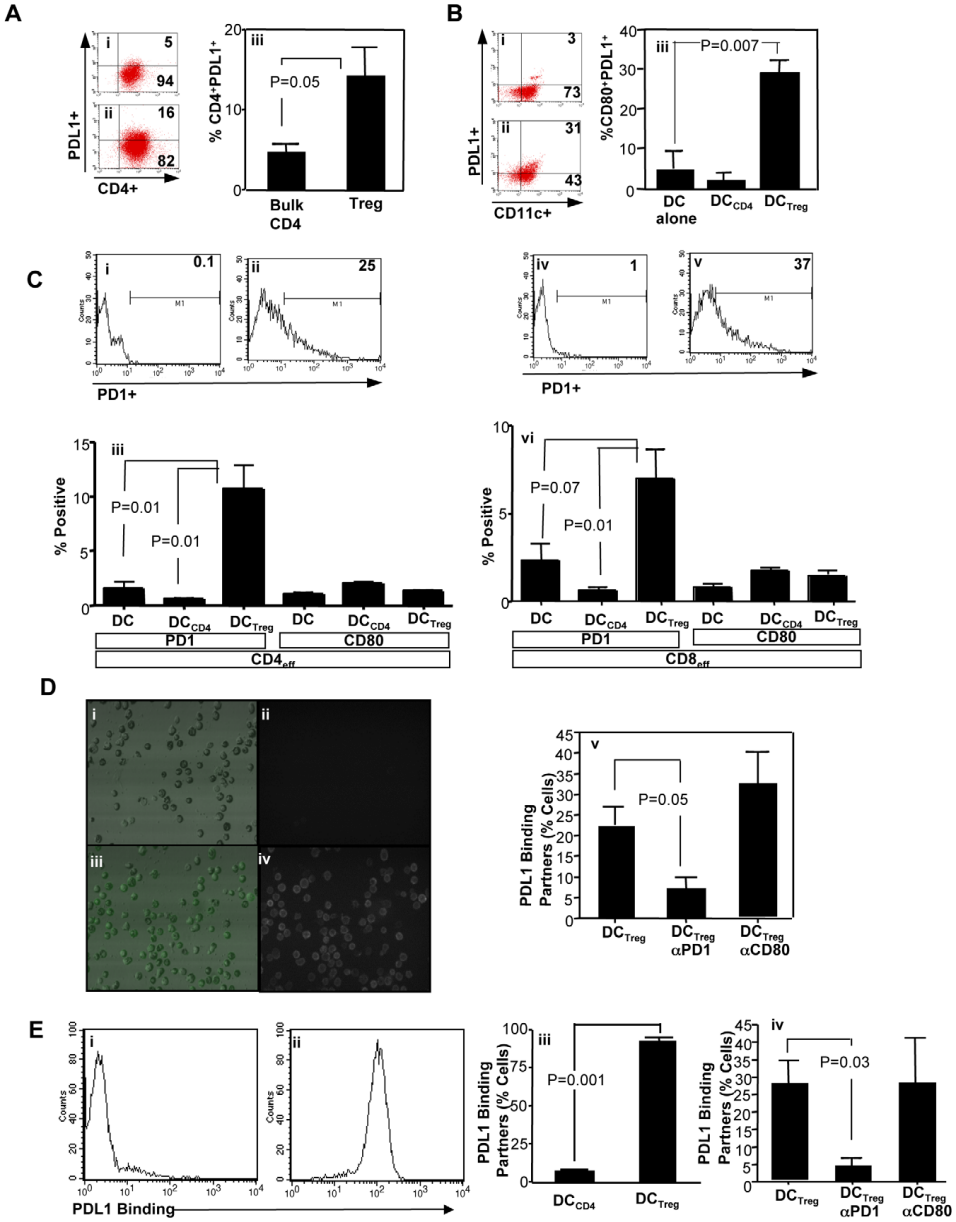
Tregs express PD-L1 and modulate the PD-1 pathway. (A) Representative flow data for control CD4 cell and Treg expression of PDL1 ((i) and (ii), respectively). (iii) represents summation of results (mean ± SEM of *n* = 4 experiments). (B) Control CD4 cells and Tregs were cocultured with pooled allogeneic DC for 24 h (Treg to DC ratio, 1∶1) to generate “DC_CD4_” and “DC_Treg_” populations, respectively. Control CD4 cells and Tregs were then removed and resultant conditioned DC were evaluated for coexpression of CD80 and PD-L1. Representative data for DC_CD4_ and DC_Treg_ conditions are shown in (i) and (ii), respectively. (iii) represents summation of results (mean ± SEM of *n* = 5 experiments). (C) Flow cytometry detection of the PD-L1 binding partners PD1 and CD80 on effector CD4^+^ cells ((i), (ii), and (iii)) and CD8^+^ cells ((iv), (v), and (vi)) after exposure to control or conditioned allogeneic DC for 48 h. Representative histograms showing isotype control (i) and PD1 staining of CD4 effectors (ii); (iii) represents summation of CD4 cell results (mean ± SEM of *n* = 3 experiments). Similarly, representative histograms showing isotype control (iv) and PD1 expression of CD8 effectors (v); (vi) represents summation of CD8 cell results (mean ± SEM of *n* = 3 experiments). (D) Laser scanning cytometry for detection of PD-L1 binding partners on both CD4+ and CD8+ responder T cells. Enriched responders were incubated with a PD-L1 fusion protein. Pseudocolor and fluorescence images of PD-L1 binding to responder T cells stimulated with control CD4 cell–conditioned DC are shown in (i) and (ii), respectively; pseudocolor and fluorescence images of PD-L1 binding to responder T cells stimulated with Treg-conditioned DC are shown in (iii) and (iv), respectively. Blocking studies were performed to determine PD-L1 receptor usage: enriched responders were blocked with anti-PD1 or anti-CD80 and then incubated with PD-L1 fusion protein (v). (E) Responder T cell binding of PD-L1 fusion protein by flow cytometry. Allo-MLR was established using control CD4- or Treg-conditioned DC. After 48 h, responder T cells were harvested, stained with PD-L1 fusion protein, and flow cytometry was performed. Representative flow histograms show responder T cell PD-L1 binding using control CD4-conditioned DC (i) or Treg-conditioned DC (ii). Pooled results from *n* = 3 normal donors are shown in (iii) (% of cells binding PDL-1; mean ± SEM); (iv) shows blocking studies for *n* = 3 donors in an independent experiment (% of cells binding PD-L1; mean ± SEM).

Further experiments were performed to characterize the mechanism of immune modulation mediated by expanded Tregs generated from CD4^+^CD127^−^ cells. Blockade of TGF-β, IL-10, IDO, CTLA4, or LAP did not abrogate Treg suppression in the allo-MLR (unpublished data). However, experiments utilizing transwell plates indicated that Treg suppression in the allo-MLR was contact dependent (unpublished data). Programmed death (PD) ligand 1 (PD-L1, or B7-H1) is expressed on DC [33], human tumor cells [34], and normal human tissue [35] and interacts with PD receptors on T cells to modulate the balance of tolerance and immunity (reviewed in [36]). In murine systems, Treg cell expression of PD-L1 associates with suppressor function [24]; in addition, endothelial cell [37] or CD8α^+^ DC [38] expression of PD-L1 promotes murine Treg generation. In humans, intratumor Tregs directly inhibited responder T cell proliferation through PD-L1 [39]. Because Tregs in our experiments expressed increased PD-L1 (Figure 1A; representative flow plot (i) and (ii); summary (iii)), we reasoned that Tregs might modulate DC via the PD-1 pathway. Indeed, allogeneic DC isolated from the Treg-containing MLR expressed increased PD-L1 relative to DC isolated from the standard MLR (Figure 1B; representative plot (i) and (ii); summary (iii)); remarkably, DC harvested from control CD4-containing MLR failed to up-regulate PD-L1. Of note, Treg-conditioned DC did not have increased expression of PD-1 (CD11c^+^PD-1^+^ cells, <1%).

PD-L1 inhibits T cell function via the PD-1 receptor and B7-1 (CD80) [40]. To determine PD-L1 binding pathways in our system, we first measured effector T cell expression of PD-1 and CD80 after incubation with three types of allogeneic myeloid DC (control, Treg conditioned, or control CD4 conditioned). Effector CD4^+^ T cells (Figure 1C; representative flow plot (i)and (ii); summary (iii)) and CD8^+^ T cells (Figure 1C representative flow plot (iv) and (v); summary (vi)) up-regulated PD-1 expression, but not CD80 expression, upon exposure to Treg-conditioned DC, but not CD4-conditioned DC. We next utilized a PD-L1 fusion protein to characterize binding pathways. Using laser scanning cytometry (LSC), we found that effector T cells up-regulated total PD-L1 binding partners in the presence of Treg-conditioned DC, but not CD4-conditioned DC (Figure 1D, left panel); importantly, effector T cell PD-L1 binding was abrogated by T cell preincubation with anti-PD1, but not anti-CD80 (Figure 1C, right panel). And finally, effector T cell PD-L1 binding was quantified by flow cytometry (Figure 1E (i)–(iii)). Remarkably, PD-L1 binding was greatly increased on effector T cells exposed to Treg-conditioned DC (% effector T cell PD-L1 binding increased from 7.3±0.4 to 92.6±2.8, *p* = 0.001); similar to results using LSC, effector T cell PD-L1 binding was abrogated by T cell preincubation with anti-PD1, but not anti-CD80 (Figure 1E (iv)).

### Allogeneic DC Conditioned with Tregs Possess Reduced Allostimulatory Capacity

**Figure 2:**
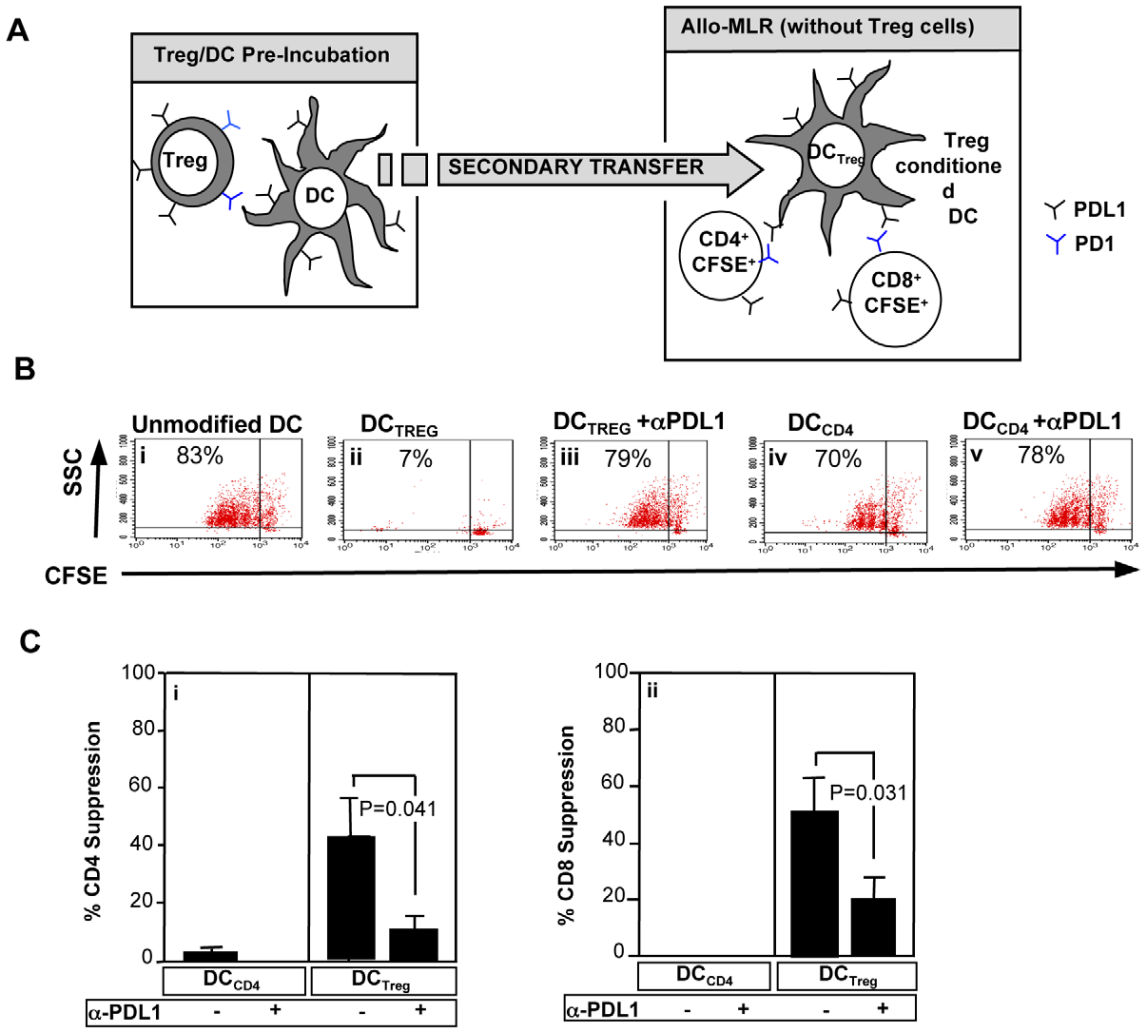
Treg-conditioned DC have reduced allostimulatory function in part through PD-L1. (A) Experimental schema for the allo-MLR using Treg-conditioned DC. Control CD4 cells or Tregs were generated ex vivo and then utilized to condition allogeneic DC (24-h incubation; 1∶1 cell ratio). Conditioned DC were then purified by negative selection using anti-CD3 microbeads and utilized as the stimulator population (DC to responder T cell ratio, 1∶20). The allo-MLR was performed in the presence of anti-PD-L1 or isotype control antibody. (B) Representative CFSE dye dilution proliferation assay results, including responder CD4 alloreactivity in response to: unmodified DC (i); DC conditioned with Tregs either without (ii) or with (iii) addition of anti–PD-L1; and DC conditioned with control CD4 cells either without (iv) or with (v) anti–PD-L1. (C) Percent inhibition of responder CD4 cell proliferation (i) and responder CD8 cell proliferation (ii) were calculated relative to proliferation measured using sham-treated DC. Results are mean ± SEM of *n* = 8 normal donors evaluated.

Secondary transfer experiments were performed to evaluate whether Tregs mediated suppression in part through DC modulation (experimental scheme, Figure 2A). Indeed, allogeneic DC conditioned with Tregs yielded reduced levels of CD4^+^ and CD8^+^ responder T cell proliferation relative to CD4-conditioned allogeneic DC (representative results, Figure 2B; pooled results, Figure 2C). Importantly, blockade of DC expression of PD-L1 partially corrected the observed stimulatory deficit of Treg-conditioned DC on CD4^+^ and CD8^+^ T cell proliferation (representative results, Figure 2B; pooled results, Figure 2C).

### Allogeneic Myeloid DC Conditioned with Tregs Modulate the PD1 Pathway In Vivo

**Figure 3:**
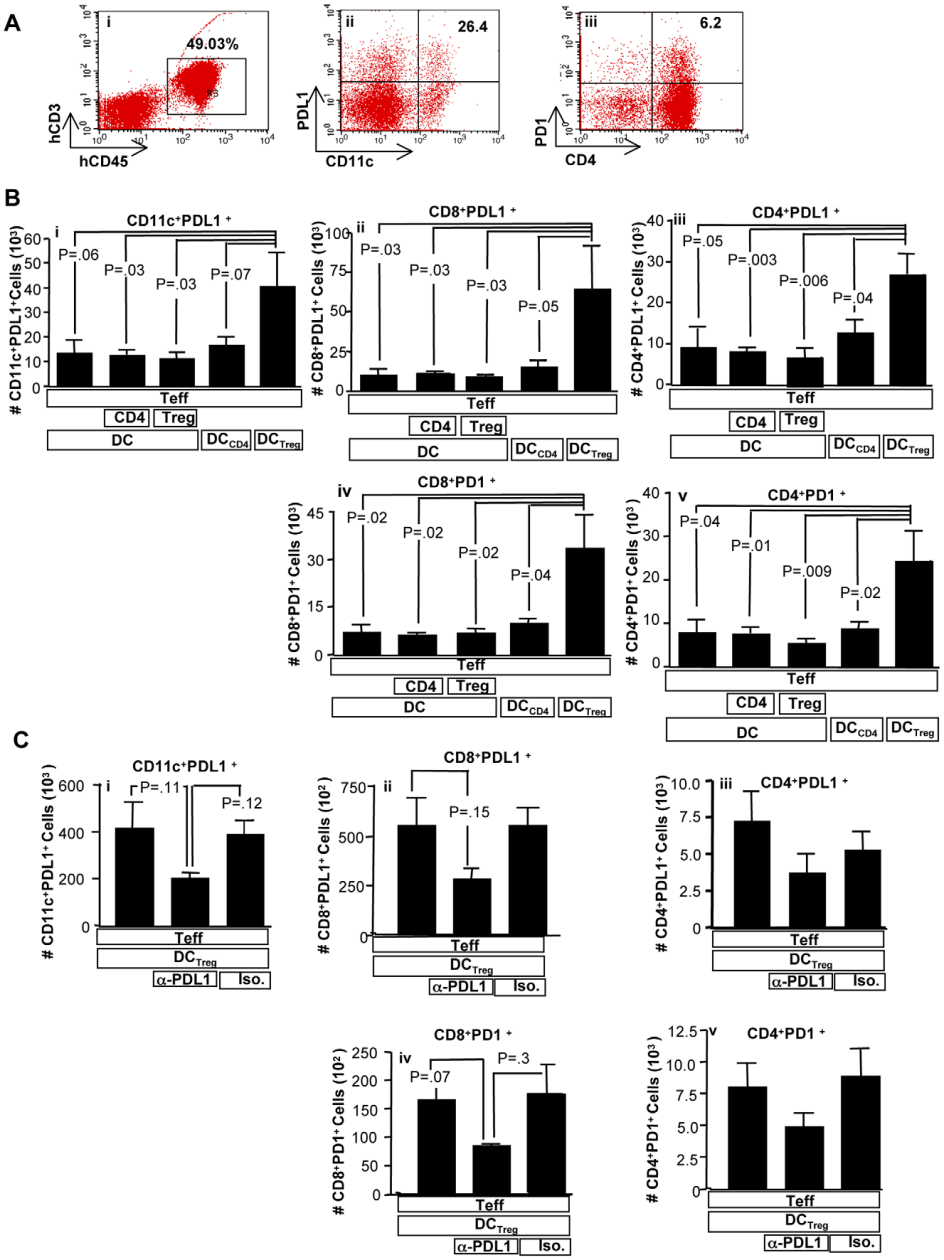
Treg-conditioned DC modulate effector T cells in vivo via PD-L1. A xenogeneic transplantation model utilized Rag2^−/−^γc^−/−^ mice that received some combination of human cells, as indicated, including: CFSE-labeled effector Teff cells (“Teff”); untreated DC (“DC”), control CD4-conditioned DC (“DC_CD4_”), or Treg-conditioned DC (“DC_Treg_”); and ex vivo–generated control CD4 cells (“CD4”) or regulatory T cells (“Treg”). (A) Spleens were harvested 24 h after cell infusion and analyzed by flow cytometry. Human cells were gated by human CD45^+^ staining including any human CD3^+^ T cells (representative data; (i)). PDL1 expression was evaluated on DC by CD11c staining (representative data, (ii)); PD1 expression was evaluated on CD4 cells (representative data, (iii)). (B) Flow cytometric analysis was used to measure the absolute number of: CD11c^+^ DC that coexpressed PD-L1 (i); CD8^+^ and CD4^+^ T cells that coexpressed PD-L1 ((ii) and (iii), respectively); and CD8^+^ and CD4^+^ T cells that coexpressed PD-1 ((iv) and (v), respectively). Results are mean ± SEM of *n* = 5 mice per cohort. (C) In a separate experiment, control CD4-conditioned or Treg-conditioned DC were incubated for 30 min with anti–PD-L1 or isotype control antibody prior to adoptive transfer; in addition, anti–PD-L1 or isotype control antibody was injected intraperitoneally immediately after cell transfer (100 µg/mouse). Spleens were harvested 24 h after cell infusion and analyzed by flow cytometry to determine the absolute number of: CD11c^+^ DC that coexpressed PD-L1 (i); CD8^+^ and CD4^+^ T cells that coexpressed PD-L1 ((ii) and (iii), respectively); and CD8^+^ and CD4^+^ T cells that coexpressed PD-1 ((iv) and (v), respectively). Results are mean ± SEM of *n* = 5 mice per cohort.

Next, we utilized an in vivo xenogeneic transplantation model to further characterize the ability of Tregs or Treg-conditioned DC to modulate the PD1 pathway. As expected, recipients of Treg-conditioned DC, which expressed increased PD-L1 in vitro prior to adoptive transfer, had an increased in vivo number of dendritic cells in the spleen that expressed PD-L1 (Figure 3A; representative flow plots (i), (ii), and (iii); summary data, 3b (i)); relative to recipients of control DC, recipients of Treg-conditioned DC also had an increase in PD-L1–expressing DC in the bone marrow (*p* = 0.006). Remarkably, recipients of Treg-conditioned DC also had increased numbers of effector CD4^+^ and CD8^+^ T cells in the spleen that expressed PD-L1 in vivo (Figures 3B (ii) and (iii), respectively); such recipients also had increased numbers of T cells that expressed PD-L1 in the bone marrow (*p* = 0.003). In marked contrast, recipients of control CD4-conditioned DC did not have increased responder T cell PD-L1 expression. Interestingly, recipients of Treg-conditioned DC also had increased numbers of effector CD8^+^ and CD4^+^ cells in the spleen that expressed PD-1 in vivo (Figures 3B (iv) and (v), respectively); in the bone marrow, such recipients also had increased numbers of CD8^+^PD-1^+^ cells (*p* = 0.02) and CD4^+^PD-1^+^ cells (*p* = 0.009).

Further experiments were performed to assess the functional significance of this sequential increase in PD-L1 expression from Treg cell, to conditioned DC, and then to responder T cells in vivo. Recipients of Treg-conditioned DC that were incubated with anti–PD-L1 prior to adoptive transfer had lower numbers of PD-L1–expressing DC in vivo, although cohort comparisons did not reach statistical significance (Figure 3C (i)); a repeat experiment yielded similar findings (unpublished data). Blockade of PD-L1 on Treg-conditioned DC yielded a reduction in the in vivo number of effector CD8 cells expressing PD-L1 (Figure 3C (ii)). Blockade of PD-L1 on Treg-conditioned DC also reduced the number of PD-L1–expressing responder effector CD4^+^ cells in the spleen (Figure 3C (iii)). Finally, PD-L1 blockade of Treg-conditioned DC reduced the in vivo number of effector CD8^+^ cells in the spleen that expressed PD-1 (Figure 3C (iv)); the number of CD4^+^PD1^+^ T cells in the spleen was not significantly altered by PD-L1 blockade (Figure 3C (v)). In sum, these data indicate that PD-L1 expression on Treg-conditioned DC was functionally significant in vivo, particularly with respect to up-regulating downstream expression of PD1 and PD-L1 on effector CD4^+^ and CD8^+^ T cells.

### Ex Vivo T Cell Activation Facilitates Human T Cell Engraftment

Next, we evaluated whether human Treg and Treg-conditioned DC might modulate xenogeneic GVHD in a PD-L1–dependent manner. Previous xenogeneic GVHD models have utilized human peripheral blood mononuclear cells (PBMCs) that contain unmanipulated human T cells [41],[42], or more recently, ex vivo costimulated T cells [43]. Our initial xenogeneic GVHD experiments utilized PBMC or purified lymphocytes as the human T cell inocula. However, despite following the protocol utilized by previous publications, we found an unacceptably low rate of lethal GVHD (<10% lethality by day 45 postinfusion); an inability to consistently generate lethality was associated with a low level of human T cell engraftment (see Figure S2A). Subsequent experiments were designed to identify human inocula that yielded enhanced human T cell engraftment and a resultant increase in lethal xenogeneic GVHD incidence. An initial experiment found that engraftment of purified human T cells was enhanced by coinfusion of a human, but not murine, source of APC (unpublished data). Based on these data, in a subsequent experiment, immune-deficient murine hosts received one of five distinct human T cell–containing inocula: (1) PBMC; (2) lymphocytes plus monocytes; (3) lymphocytes plus DC; (4) ex vivo–activated effector T cells plus monocytes; and (5) ex vivo–activated T cells plus DC. At day 30 postinfusion, recipients of the ex vivo–activated T cells plus DC had the highest levels of human CD4^+^ and CD8^+^ T cell engraftment (Figure S2A (i) and (ii), respectively); furthermore, recipients of ex vivo–activated T cells plus DC had the highest capacity for secretion of human IFN-γ at day 30 postinfusion (Figure S2B). Therefore, in order to evaluate the effects of Tregs, Treg-conditioned DC, and the PD-1 pathway in a more stringent model of xenogeneic GVHD, subsequent experiments utilized human inocula that contained ex vivo–activated T cells and DC.

### Tregs or Treg-Conditioned DC Limit Human T Cell Number and Function In Vivo

**Figure 4:**
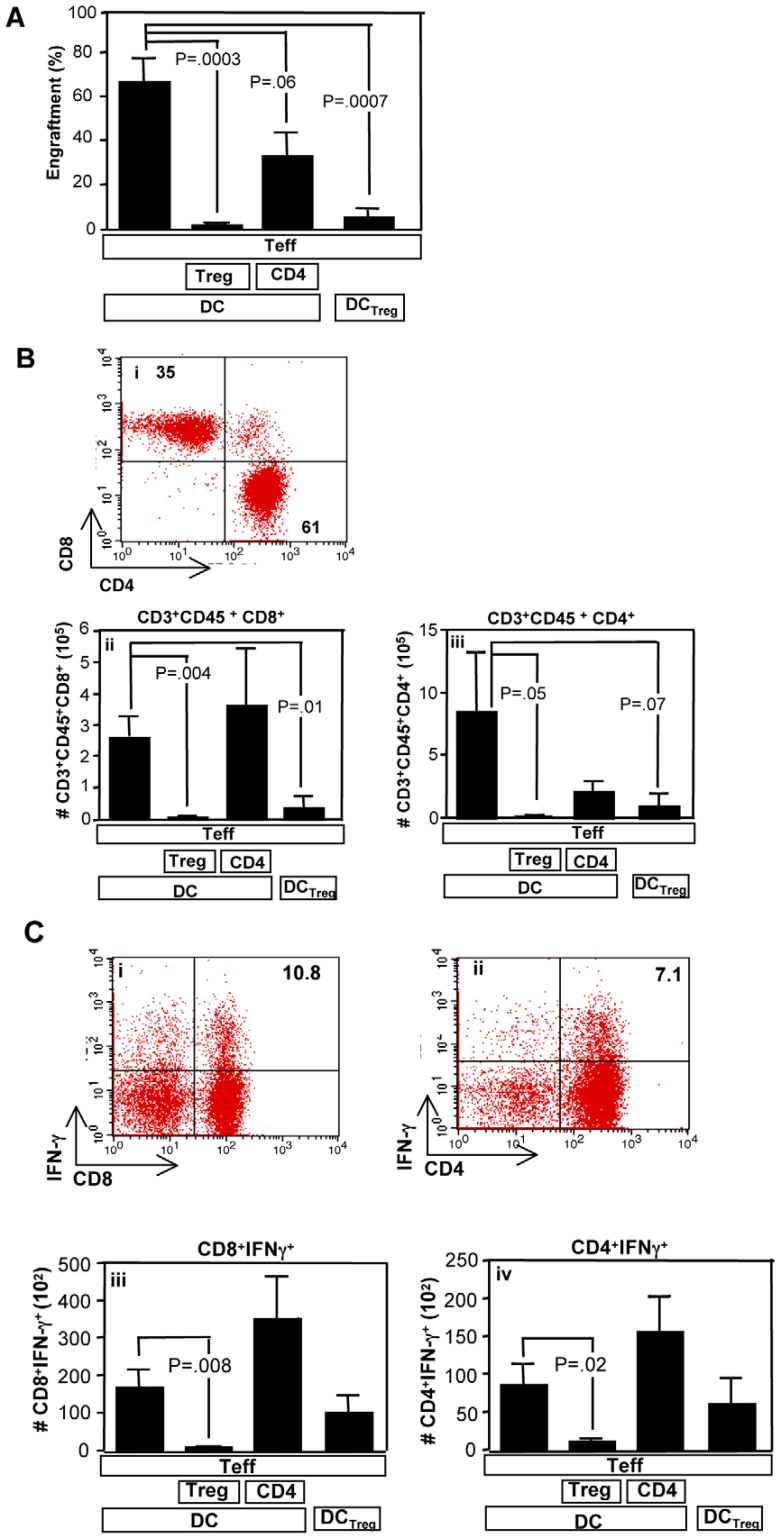
Reduction in human effector T cell numbers in vivo by Tregs or Treg-conditioned DC. Rag2^−/−^γc^−/−^ mice were reconstituted with human cells, as indicated, including: effector Th1/Tc1 cells (“Teff”); untreated DC (“DC”) or Treg-conditioned DC (“DC_Treg_”); and ex vivo–generated control CD4 cells (“CD4”) or regulatory T cells (“Treg”). Teff, DC, and Treg doses were 1×10^7^, 0.5×10^6^, and 0.5×10^6^ cells per recipient, respectively. One additional cohort received Teff cells in combination with control CD4-conditioned DC; this cohort is not shown because of early posttransplant lethality due to xenogeneic GVHD. (A) Spleens were harvested on day 45 posttransplant and percent human cell engraftment was determined by flow cytometry. (B) Representative flow data of human CD4^+^ and CD8^+^ T cell engraftment (i). Summation data for the absolute number of human CD8^+^ T cells engrafted in the spleen (ii) and human CD4^+^ T cells engrafted in the spleen (iii). (C) At day 45 posttransplant, splenocytes were costimulated for 24 h, and IC flow cytometry was performed to detect human CD4^+^ and CD8^+^ T cells capable of IFN-γ secretion. Representative flow results are shown in (i) and (ii). Summation results for determination of the absolute number of human CD4^+^IFN-γ^+^ cells and CD8^+^IFN-γ^+^ cells per spleen are shown in (iii) and (iv), respectively. All results are mean ± SEM for *n* = 5 mice per cohort.

Further in vivo experiments were performed to evaluate the effect of Tregs and Treg-conditioned DC on human T cell engraftment, cytokine activation, and induction of lethal xenogeneic GVHD. Recipients of human inocula that contained either Tregs or Treg-conditioned DC had reduced absolute numbers of human T cells as measured in the spleen at day 45 posttransplant (Figure 4A); the absolute number of human T cells present in vivo was also reduced when the evaluation was performed in the bone marrow for recipients of both Tregs (*p* = 0.03) and Treg-conditioned DC (*p* = 0.01). Tregs and Treg-conditioned DC transfer resulted in reduced absolute numbers of both human effector CD8^+^ and CD4^+^ cells (Figure 4B; representative data (i); summation of data in (ii) and (iii), respectively). Human CD4^+^ T cell numbers in the bone marrow was also reduced for recipients of Tregs (*p* = 0.01) but not significantly reduced in recipients of Treg-conditioned DC (*p* = 0.08); human CD8^+^ T cell numbers in the bone marrow were also reduced for recipients of Tregs (*p* = 0.02) but not significantly reduced in recipients of Treg-conditioned DC (*p* = 0.09). Of note, recipients of Tregs, but not recipients of Treg-conditioned DC, had a statistically significant reduction in the absolute number of posttransplant CD8^+^Tc1 and CD4^+^Th1 cells capable of IFN-γ secretion (Figure 4C (i) and (ii), representative flow plots; 4C (iii) and (iv), summation of data). In sum, these data indicated that both Treg cells and Treg-conditioned DC were capable of inhibiting human T cells in vivo, with Treg therapy manifesting more potent regulation both in terms of limiting T cell numbers and T cell effector function.

### Tregs or Treg-Conditioned DC Protect against Lethal Xenogeneic GVHD

**Figure 5:**
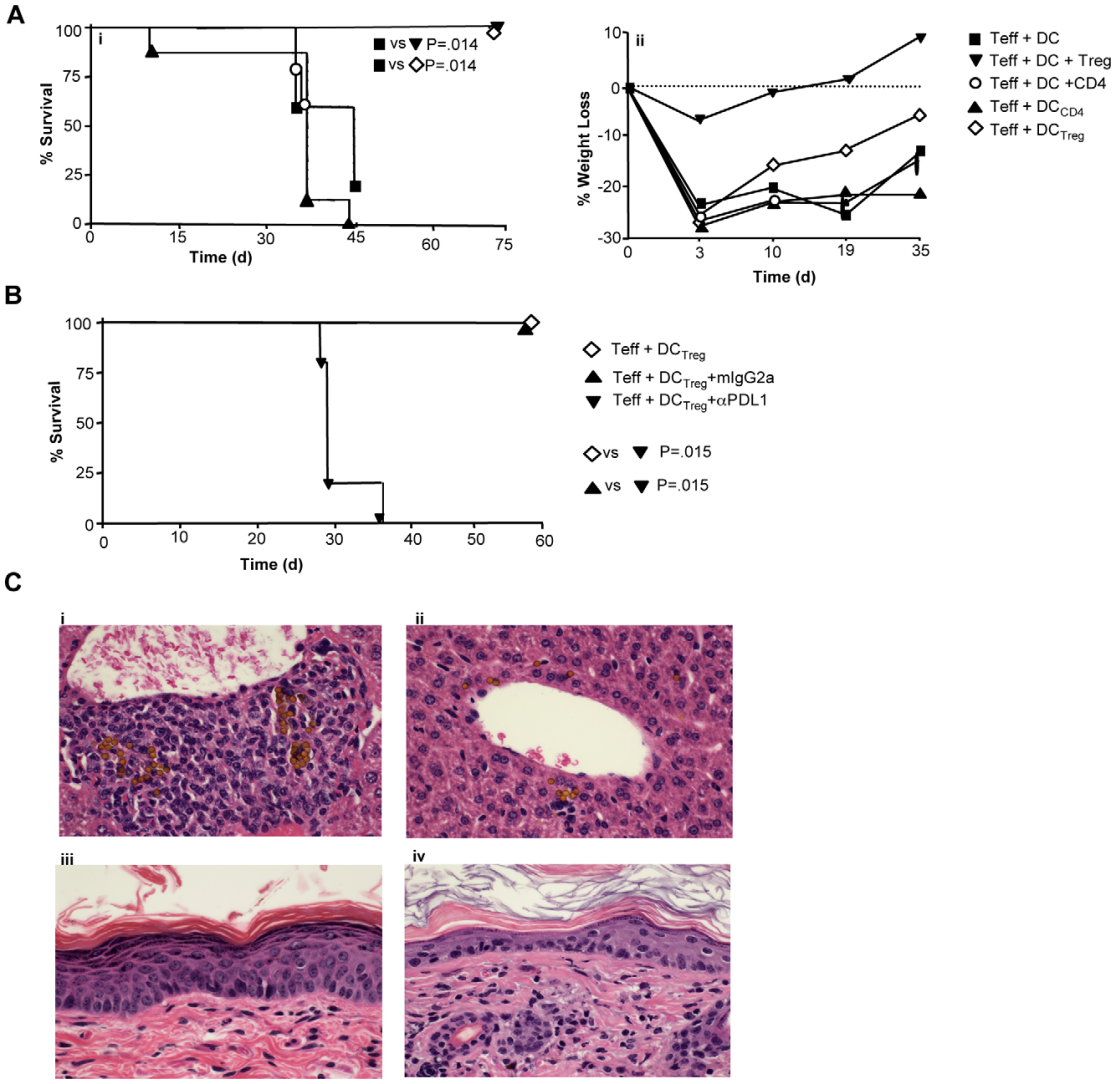
Tregs or Treg-conditioned DC protect against lethal xenogeneic GVHD. Using Rag2^−/−^γc^−/−^ mice as host, transplant cohorts received effector T cells (“T_eff_”) in combination with allogeneic DC, control CD4 cell-conditioned DC (“DC_CD4_”), or Treg-conditioned DC (“DC_Treg_”); other cohorts received Teff cells in combination with allogeneic DC plus either Tregs (“Treg”) or control CD4 cells (“CD4”). The doses of the Teff, DC, and Treg cells were 3×10^7^, 3.0×10^6^, and 1.5×10^6^ cells per recipient, respectively. (A) Overall survival is shown in (i); posttransplant weight loss is shown in (ii). (B) In an independent experiment, transplants were performed at these same cell doses, and posttransplant survival was determined. Treg-conditioned DC were incubated with anti–PD-L1 (“αPDL1”) or isotype control antibody (“mIgG2a”) for 30 min prior to adoptive transfer; in addition, anti–PD-L1 or isotype control antibody was injected i.p. immediately after cell transfer (100 µg/mouse). (C) Representative result of histology analysis performed at day 30 posttransplant demonstrates T cell infiltration of liver in the GVHD control group (i) and minimal infiltration in recipients of Tregs (ii). Representative histology of skin demonstrates cutaneous acanthosis and hyperkeratosis in GVHD controls (iii) and minimal skin pathology in recipients of Tregs (iv).

Xenogeneic GVHD was evaluated by weight loss measurement, survival analysis, and histology evaluation of GVHD target tissues. Recipients of Tregs or Treg-conditioned DC were uniformly protected against lethal xenogeneic GVHD (Figure 5A (i)); importantly, recipients of control CD4-conditioned DC uniformly died of xenogeneic GVHD. Posttransplant weight loss, which is a more sensitive clinical parameter of xenogeneic GVHD, was moderated by Treg-conditioned DC therapy and virtually eliminated by Treg therapy (Figure 5A (ii)). In a second experiment, we confirmed the ability of Treg-conditioned DC to completely abrogate the generation of lethal xenogeneic GVHD; importantly, protection against lethal xenogeneic GVHD conferred by the Treg-conditioned DC was completely abrogated by anti–PD-L1, but not by isotype control antibody (Figure 5B). Of note, both control DC and Treg-conditioned DC engrafted and persisted in vivo; importantly, such numbers were not substantially influenced by Treg therapy or anti-PDL1 antibody. That is, at day 25 posttransplant, the absolute numbers of CD11c^+^ DC per spleen (each value, ×10^3^; *n* = 5 per cohort) in transplant recipients that received effector human T cells in combination with the indicated specific type of human DC were 136±11 (control DC), 107±6 (control DC and Treg therapy), 418±98 (Treg-conditioned DC), 163±63 (Treg-conditioned DC, anti–PDL1-treated), and 279±77 (Treg-conditioned DC, isotype antibody treated) (each comparison, *p* = NS by ANOVA test). GVHD control mice uniformly developed a diffuse skin rash and hair loss; skin histology analysis documented cutaneous acanthosis and hyperkeratosis in GVHD controls, but not in Treg recipients (representative histology; Figure 5C (iii) and (iv), respectively). Furthermore, GVHD controls, but not Treg recipients, developed diffuse lymphocytic infiltration of the liver (representative histology; Figure 5C (i) and (ii), respectively).

## Discussion

The rational design of adoptive cell therapy protocols using ex vivo–expanded Tregs would be facilitated by an improved understanding of their cellular and molecular mechanism of action, which has been difficult to ascertain, particularly with respect to human Tregs. In this report, utilizing a novel method of generating human Tregs based on CD127 negative selection, we have elucidated a unique Treg mechanism of immune suppression analogous to previously described models of infectious tolerance [44] that is mediated at least in part by modulation of allogeneic dendritic cells through the PD-L1 pathway. This mechanistic understanding is particularly pertinent to efforts that will utilize Tregs for the prevention or treatment of GVHD, which is driven by allogeneic DC [30] and is amenable to suppression through PD-1 [45].

Our results are the first, to our knowledge, to describe a mechanism of human Treg action that involves potent in vitro and in vivo suppression of effector T cells through a secondary cellular messenger, myeloid dendritic cells. A similar biology has been described in murine models, whereby Tregs create a weak stimulator DC through induction of immunosuppressive indoleamine 2,3-dioxygenase (IDO) via a CTLA-4– or IFN-γ–dependent pathway [26]. Interestingly, the reverse biology has also been described in murine models, whereby murine plasmacytoid DC that produce IDO promote the generation of immunosuppressive Tregs that express PD-L1 [24]. Of note, in our experiments, inhibition of IDO by 1-MT treatment did not abrogate suppression mediated by Treg-conditioned myeloid DC (unpublished data). Similar to a previous study using freshly isolated human Tregs [29], we found that ex vivo–generated human Tregs inhibited myeloid DC secretion of the proinflammatory cytokines IL-6 and TNF-α (unpublished data) and induced a DC phenotype with greatly reduced capacity to induce responder T cell proliferation in vitro. Most importantly, we have significantly extended this prior work through our discovery that myeloid DC conditioned by Tregs were effective in vivo for the complete elimination of posttransplant lethal xenogeneic GVHD induced by effector T cells. In addition to this apparent DC-mediated mechanism of GVHD protection, other non-APC mechanisms are likely operative for the Tregs that we studied, because transplant cohorts that received Tregs had more robust protection against xenogeneic GVHD than recipients of Treg-conditioned DC (lowest CD4^+^ and CD8^+^ T cell engraftment, lowest posttransplant IFN-γ secretion, and lowest degree of weight loss posttransplant).

Furthermore, this is the first demonstration that human Tregs mediate immune suppression in vivo through modulation of the PD-1 pathway. First, we observed that ex vivo–expanded human Tregs expressed increased PD-L1 relative to control expanded CD4^+^ T cells. Second, Treg-conditioned DC expressed greatly increased PD-L1 relative to DC conditioned with control CD4 cells. As such, Treg PD-L1 appeared to directly induce DC PD-L1 expression; the potential existence of such a PD-L1 “positive feedback loop” adds to the known complexity of PD-1 pathway regulation [36] and to our knowledge has not been previously described for murine or human Tregs. Third, this feedback appeared to extend to the distal stage of effector T cell regulation because effector CD4^+^ and CD8^+^ cells under the influence of Treg-conditioned DC, but not control CD4-conditioned DC, had nearly universal expression of PD-L1 binding partners. Finally, we determined that such effector T cell binding to PD-L1 was preferentially mediated through PD-1 rather than the other receptor associated with this pathway, CD80. It is interesting to note that a recent study found that PD-1 expression on Treg cells in patients with viral hepatitis played a negative regulatory role for Treg cell function via limitation of STAT-5 phosphorylation [46]. In our experiments, the ex vivo–activated Treg cells expressed a high level of PD-1, yet were able to mediate potent suppression of effector T cells in vivo at relatively dilute Treg to effector T cell ratio; as such, it does not appear that the PD-1 pathway exerted a functionally significant down-regulatory effect on the Tregs utilized in our model. It is interesting to note that the Treg-conditioned DC did not express significant PD-1; it is thus possible that the capacity of this cell population to effectively prevent xenogeneic GVHD may reside in part on a limited susceptibility to PD-1–mediated suppression.

Importantly, this biology was functional in vivo because: (1) Treg-conditioned DC maintained expression of PD-L1 after adoptive transfer; (2) effector (Teff) cells up-regulated both PD-L1 and PD1 in vivo in the presence of Treg-conditioned DC; and (3) a significant proportion of this immune modulation was abrogated if Treg-conditioned DC were blocked with anti–PD-L1. Remarkably, the survival advantage conferred by Treg-conditioned DC was fully abrogated by anti–PD-L1. In sum, these data demonstrate that modulation of the PD-1 pathway represents a significant mechanism of action of ex vivo–expanded Tregs that involves an interaction between Tregs, DC, and effector T cells in an apparent positive feedback loop. Further experiments will be required to better understand this process of intercellular PD-1 pathway modulation. Potentially, the PD-L1 suppressor phenotype might be transferred from Tregs to DC and then to effector T cells by a process of trogocytosis [47], which results in the generalized transfer of cell membrane proteins, including costimulatory molecules [48]. However, because we observed that Tregs up-regulated DC expression of PD-L1, but not other cell surface molecules such as PD-1, we speculate that alternative mechanisms of intercellular regulation may be operational.

These findings have several implications for ongoing efforts to utilize ex vivo–generated Tregs for adoptive cell therapy. First, we have found that CD127^−^ selection represents a suitable alternative to CD25^+^ selection for attempts to enrich for Tregs prior to ex vivo expansion; further experiments will be required to directly compare these two methodologies to determine whether such methods result in differential modulation of APC function through the PD-1 pathway. It is perhaps important to emphasize that the regulatory T cell or Treg-conditioned DC modulation of xenogeneic GVHD was robust because it occurred at the relatively low regulatory cell to effector cell ratio of 1∶20, which is considered to represent a physiologic ratio. Second, our demonstration that ex vivo–generated Tregs operate to a significant degree indirectly through allogeneic myeloid DC may help guide protocol design, particularly in the setting of allogeneic HSCT. One theoretical limitation to Treg cell therapy is the transfer of “contaminating” effector T cells or the conversion of Tregs to proinflammatory Th17 cells [49] that are known to induce GVHD [10]. The APC mechanism we have identified offers a solution to this potential limitation: that is, one could harvest host-type monocytes pretransplant and generate myeloid DC in a manner similar to the methods that we utilized, condition such DC with ex vivo–generated Tregs, and then transfer only the conditioned host DC prior to allogeneic HSCT. Such an approach would be analogous to that proposed for type II DC (DC2 cells) that promote Th2 cytokines and prevent murine GVHD [50]. Our results also indicate that the capacity of adoptively transferred Tregs to modulate GVHD may relate in part to the bioavailability of host-type myeloid DC. This consideration may have relevance to the choice of host conditioning for Treg protocols: predictably, non-myeloablative regimens may be favorable in this regard because such regimens would preserve host myeloid DC as a key secondary cellular mediator of the Treg therapy.

It should be stated that xenogeneic models of GVHD likely do not fully reflect the biology of clinical GVHD, and as such, the potential clinical implications of the findings in our model must be interpreted with caution. Specifically, the xenogeneic transplantation model that we utilized did not incorporate a human hematopoietic stem cell component, and as such, the potential effect of the regulatory T cells or Treg-conditioned DC that we evaluated on stem cell engraftment was not assessed. However, we found that human dendritic cell and effector T cell engraftment was persistent at the relatively late time points of day 25 and day 45 posttransplant, respectively; thus, it is possible that human hematopoietic progenitor cell engraftment would also be durable under the conditions that we evaluated. Such a possibility would be consistent with data emanating from murine models of MHC-disparate transplantation, which have found Treg adoptive transfer to augment allogeneic hematopoietic stem cell engraftment [7],[51]. Our findings relating to PD-1 pathway modulation may also hold clinical implications. Adoptive cell therapy using Tregs or Treg-conditioned DC may be conceptualized as a vehicle for PD-L1 delivery. Such a cell therapy approach may have immediate practical benefit for the treatment of the myriad of diseases that may benefit from PD-1 agonism [52]. That is, although an antibody-based method of PD-1 antagonism has already been investigated in phase I clinical trials [53], it is unclear whether agonistic PD-1 antibodies will be available or safely administered. Finally, the mechanisms we have identified will provide a rationale for monitoring PD-1 and PD-L1 expression on posttransplant T cells and DC as a biological marker for in vivo activity of the administered Tregs or Treg-conditioned DC; in addition, surface PD-L1 expression may be utilized as a marker to facilitate a functionally defined release criteria for the experimental cell therapy products.

In conclusion, ex vivo expansion of CD127-negatively selected CD4^+^ T cells yielded a human Treg product that inhibited alloreactivity in vitro and in vivo, in large part due to modulation of myeloid DC and a multifaceted promotion of the PD-1 pathway in Tregs, DC, and effector T cells. As such, we have identified two distinct cell therapy vehicles, Tregs and Treg-conditioned myeloid DC, each of which show promises as a novel approach to modulate human effector T cells through the PD-1 pathway.

## Materials and Methods

### Mice

Female RAG2^−/−^γc^−/−^ mice were obtained from Taconic and utilized at 8–12 wk of age. Experiments were performed according to a protocol approved by the National Cancer Institute Animal Care and Use Committee. Mice were housed in a sterile facility and received sterile water and pellets. As in previously reported methods [31],[41], mice were injected with 0.1 ml of chlodronate-containing liposomes (Encapsula Nanoscience) for macrophage depletion and given low-dose irradiation (350 cGy).

### Antibodies and Reagents

X-VIVO 20 media was obtained from BioWhitaker and AB serum was from Gem Cell. CD4 microbeads were from Miltenyi Biotec. Sheep anti-mouse (SAM) IgG dynabeads were from Dynal. Anti-CD3, anti-CD28 coated tosyl-activated magnetic beads were manufactured as previously described [54]. Rapamycin was from Wyeth (Rapamune). Recombinant human (rh) IL-2 and rhIL-12 were from PeproTech, and rhTGF-β1, αTGF-β1, -β2, -β3, and purified αPD-L1 were from R&D Systems. All other antibodies (unless otherwise stated) were provided by BD Biosciences; anti-human Foxp3 APC was from eBioscience. Luminex kits for detection of IFN-γ and TNF-α were from Bio-Rad. 5-(and-6)-Carboxyfluorescein diacetate, succinimidyl ester [5(6)-CFDA, SE; CFSE] was from Invitrogen.

### T Cell Subset Isolation

Normal donor peripheral blood cells were collected by apheresis on an IRB-approved protocol. Total lymphocytes were isolated by elutriation [55]. Total CD4^+^ T cells were then enriched by CD4 microbeads according to manufacturer instructions. To isolate CD127-depleted CD4^+^ T cells: (1) elutriated lymphocytes were adjusted to 100×10^6^ cells/ml and incubated with anti-CD127 (10 µg/ml, 30 min, 4°C); (2) cells were washed, mixed with SAM dynabeads (bead∶cell ratio, 4∶1), incubated (30 min, 4°C), separated (hand-held magnet, Dynal); and (3) CD127-depleted cells were subjected to CD4 cell isolation by microbeads.

### Ex Vivo Culture of Total CD4^+^ and CD4^+^CD127^−^ T Cell Subsets

Total CD4^+^ and CD4^+^CD127^−^ T cells were cultured in polystyrene tissue culture flasks (Corning). Cells were activated by anti-CD3, anti-CD28 costimulation (bead∶cell ratio, 3∶1), and cultured in X-VIVO 20 with 5% heat-inactivated (HI) AB serum containing rapamycin (1 µM), TGF-β1 (20 ng/ml), rhIL-2 (100 IU/ml). rhIL-2 alone was added at days 2, 4, and 6. Cultures were started at 1.5×10^6^ cells/ml, maintained at 1×10^6^ cells/ml through day 7, and then split daily to 0.5×10^6^/ml by addition of IL-2 and rapamycin-replete medium through day 12.

### Flow Cytometry

T cells were washed with PBS supplemented with 0.1% BSA and 0.01% azide, and stained using anti-: CD4 PE-cy7 (clone S3.5; Caltag), Foxp3 APC (clone 249D; eBioscience), CCR7 PE (clone 150503; R&D), CTLA-4 Biotin (clone BN13), CD27 FITC (clone M-T271), and CD62L APC-cy7 (clone DREG-56; Biolegend). For intracellular (IC) flow cytometry, fixation and permeabilization buffer was utilized (eBioscience); four-color IC flow cytometry was performed with combinations of anti-: IL-2 biotin (clone B33-2), IFN-γ APC (clone B27), CD4 Pe-Cy5 (clone RPA-T4), and Foxp3 PE (clone PCH101; eBioscience). DC were evaluated using anti-: CD80 Bio (clone L307.4), CD86 APC (clone 2331), CD14 PE (clone M5E2), CD83 FITC (clone HB15e), CD40 APC (clone 5C3), and PDL1 PE-cy7 (clone MIH1).

### Generation of Myeloid DC

Monocytes from four healthy, randomly selected donors were obtained by apheresis and elutriation; HLA typing confirmed that the donors did not share major haplotypes. Each monocyte population was cultured in X-VIVO 20 medium with 5% HI-AB serum, rhGM-CSF (50 ng/ml), and rhIL-4 (20 ng/ml). On day 5, each DC culture was enumerated and subjected to flow cytometry to document a DC phenotype (CD14^−^, CD11c^+^, CD83^+^, CD80^+^, CD86^+^; unpublished data). The four separate DC populations were pooled in equal proportions, and aliquots of the final product were cryopreserved and utilized for each experiment.

### Allogeneic Mixed Lymphocyte Reaction (MLR)

Normal donor lymphocytes (“responder T cells”; 2×10^5^ cells) were cocultured with allogeneic DC (5×10^4^ cells) in 96-well round-bottom plates (T cell to DC ratio, 20∶1). To detect proliferation, responder T cells were CFSE-labeled before coculture. From the same normal donors, Tregs were generated from CD4^+^CD127^−^ cells, or as a control, from total CD4^+^ cells. Initial experiments determined that a Treg to responder T cell ratio of 1∶20 consistently yielded suppression of proliferation. Proliferation of CD4^+^ and CD8^+^ responder T cells was evaluated by CFSE dye dilution; percent suppression of CD4 and CD8 responder T cell suppression was calculated, with values representing the ratio of total divided peaks to both divided and nondivided peaks, normalized to the sham-treated experimental group.

### Mechanistic Assays Relating to the MLR

During the MLR, neutralizing antibodies were added, including anti-: CTLA-4, IL-10, TGF-β1, TGF-β2, TGF-β3, LAP, and their respective isotope controls. All antibodies were used at 20 µg/ml. A combination of anti-CTLA-4, anti-TGF-β, and anti-LAP was also tested. 1-methyl-d-tryptophan (1-MT, 1 mM; Sigma) was utilized to inhibit indoleamine 2,3-dioxygenase (IDO). Transwell plates with a 4-mm membrane (Corning LifeSciences) were utilized to assess Treg contact dependency.

### Isolation of Treg-Conditioned Dendritic Cells

For the secondary transfer experiments, DC were incubated with Tregs for 48 h (Treg to DC ratio, 1∶1). Tregs were then removed using T cell–positive selection (anti-CD3 microbeads and subsequent magnetic column separation; Miltenyi); the resultant population was >99% pure for DC content, as determined by flow cytometry using CD11c in combination with CD80, CD86, and CD40. Such Treg-conditioned DC were then used as stimulator cells, with degree of proliferation determined relative to DC conditioned with control T cells (cells generated ex vivo from total CD4 cells) or sham-treated DC. MLR assays using preconditioned DC were also performed with anti–PD-L1 (20 µg/ml) or isotype control antibody.

### Laser Scanning Cytometry

On day 5, responder T cells were evaluated for expression of PD-L1 binding partners PD1 and CD80. The responder T cells were blocked with a specific αPD1 (1 µg/1×10^6^ cells) and αCD80 (1 µg/1×10^6^ cells) antibody and then PD-L1 binding was studied by incubation with recombinant PD-L1-Fc fusion molecule (R&D); secondary incubation was performed with FITC-labeled rabbit anti-human IgG, Fc-fragment antibody (Jackson Laboratory). Stained T cells were delivered to 96-well plates with a plastic #1 cover slip bottom (1×10^5^ cells in 200 µl) and analyzed (iCys Laser Scanning Cytometer; Compucyte Corporation). Cells were scanned (488-nm laser) and fluorescence was detected (530/30-nm band-pass filter). Scan images and fluorescence data were generated (iGeneration and innovator software; Compucyte). Images were collected at 0.5-µm scan resolution.

### Xenogeneic GVHD Model

Human effector CD4^+^Th1/CD8^+^Tc1 (Teff) cells were generated by T cell culture for 6 d by costimulation and expansion of T cells in rhIL2 (20 IU), αIL-4 (100 ng/ml), rhIL-12 (20 ng/ml), and rapamycin (1 µM). On day 6 of culture, Teff cells were harvested and injected (i.v. by retro-orbital method, as previously described [43]) into Rag2^−/−^γc^−/−^ mice conditioned with chlodronate and radiation; Teff cell dose was either 1 or 3×10^7^ cells/recipient (higher dose used for evaluation of posttransplant lethality). Specific cohorts additionally received ex vivo–generated Tregs (generated from CD4^+^CD127^−^ cells) or control T cells (generated from total CD4 cells) at a dose of 0.5 or 1.5×10^6^ cells/recipient such that the in vivo ratio of effector Teff cells to Tregs always matched that utilized in the allogeneic MLR (20∶1). As indicated, cohorts additionally received pooled allogeneic DC (complete mismatch as compared to Teff and Tregs) utilized in the MLR (DC dose, 0.5 or 1.5×10^6^ cells/recipient) to maintain constant ratios; allogeneic DC were either not conditioned or conditioned with ex vivo–generated Tregs or control T cells. For blocking experiments, conditioned DC were incubated with anti-PD-L1 (20 µg/ml) or isotype control antibody prior to adoptive transfer. In some cases, anti-PD-L1 was injected following cell transfer (i.p.; 100 µg/recipient). After adoptive transfer, human engraftment was calculated using flow cytometry data from splenic single-cell suspensions (% huCD45^+^ = [huCD45^+^ (huCD45^+^+ mCD45^+^)]×100%). Surface or intracellular flow cytometry was performed at indicated days after adoptive transfer to assess in vivo modulation of responder human CD4 and CD8 T cells and human DC.

### Statistical Analysis

Flow cytometry and cytokine data were analyzed using Student 2-tailed *t*-tests. Comparison values of *p*<0.05 were considered statistically significant. Survival was determined using Kaplan-Meier test. For three pairwise cohort comparisons, statistical analyses was performed using the Holm method [56].

## Supporting Information

Figure S1 **Ex vivo T cell expansion and day 12 phenotype.** Ex vivo costimulation and expansion in medium containing IL-2, rapamycin, and TGF-β was performed on total bulk CD4^+^ input cells or CD4^+^CD127^−^ input cells to generate control CD4 cells (“bulk CD4”) and regulatory T cells (“Treg”), respectively (results from *n* = 8 normal donors). (A) Fold-expansion from day 0 to day 12 of culture. (B) At day 12, cells were evaluated for coexpression of CD62L and CCR7 (B)(i) and Foxp3 expression (B) (ii). (C) Bulk CD4 cells and Tregs were restimulated for 24 h, and IC flow cytometry was performed to evaluate effector T cell coexpression of Foxp3 and IL-2 (i) or IFN-γ (ii). Results are mean ± SEM of *n* = 5 cultures. (D) Control CD4 cells and Tregs were compared for ability to suppress CD4^+^ (i) and CD8^+^ (ii) T cell proliferation over 5 d of culture in response to allogeneic DC (mean ± standard error of the mean [SEM] of *n* = 8 donors). (E) Representative data showing control CD4 and Treg suppression of responder CD4 (i) and CD8 (ii) proliferation over a range of suppressor cell to target ratios of 1∶20 to 1∶100.
(0.22 MB TIF)

Figure S2 **Human T cell numbers in vivo: role of ex vivo T cell activation.** Rag2^−/−^γc^−/−^ mice were reconstituted with human cells, as indicated, including: PBMC alone (“PBMC”), purified lymphocytes plus either monocytes (“Lymph+mono”) or DC (“Lymph+DC”), or ex vivo-expanded effector T cells alone plus either monocytes (“Teff+mono”) or DC (“Teff+DC”). The dose of effector T cells and APC populations were 1×10^7^ and 0.5×10^6^ cells per recipient, respectively. (A) On day 30 after transplant, the number of human CD4^+^ and CD8^+^ T cells in the spleen was determined using flow cytometry ((i) and (ii), respectively). (B) At day 30 posttransplant, splenic cells were costimulated using anti-human CD3 and CD28 beads; the resultant 24-h supernatant was then tested for content of IFN-γ by multiplex bead array. All results shown are the mean ± SEM of *n* = 10 recipients per cohort. An asterisk (*) indicates that the difference relative to the PBMC cohort was statistically significant (*p*<0.05).
(0.14 MB TIF)

## Abbreviations

allo-MLR: allogeneic mixed lymphocyte reaction
APC: antigen-presenting cell
DC: dendritic cell
GVHD: graft versus host disease
PBMC: peripheral blood mononuclear cell
Treg: regulatory T cell
