# Supplementary figures and images for: Regulatory T Cells and Human Myeloid Dendritic Cells Promote Tolerance via Programmed Death Ligand-1
Source: PLoS Biol. 2010 Feb 2;8(2):e1000302. doi: 10.1371/journal.pbio.1000302 (PMC2814822; doi:10.1371/journal.pbio.1000302)

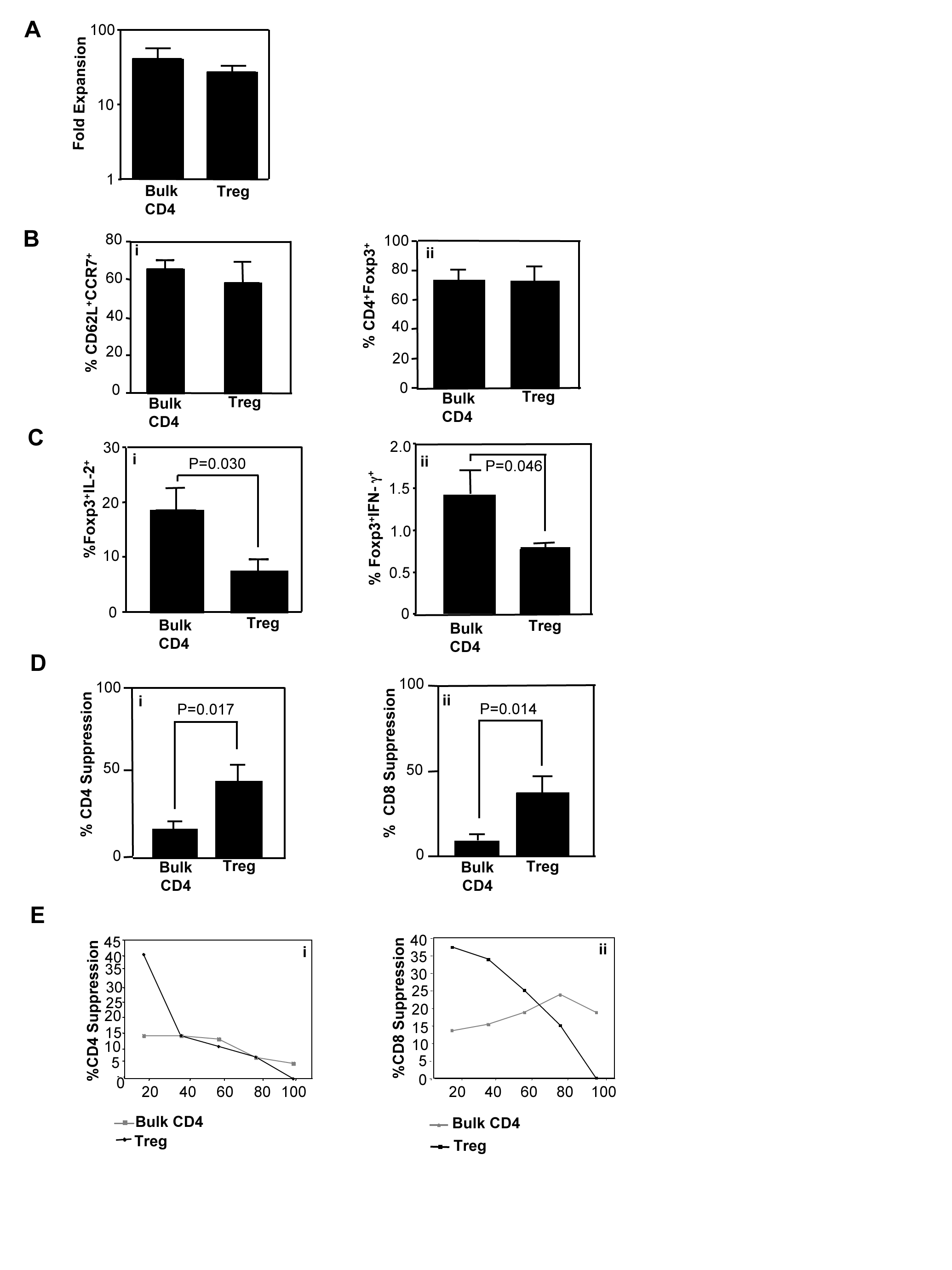

Supplement: Figure S1 — Ex vivo T cell expansion and day 12 phenotype. Ex vivo costimulation and expansion in medium containing IL-2, rapamycin, and TGF-β was performed on total bulk CD4+ input cells or CD4+CD127− input cells to generate control CD4 cells (“bulk CD4”) and regulatory T cells (“Treg”), respectively (results from n = 8 normal donors). (A) Fold-expansion from day 0 to day 12 of culture. (B) At day 12, cells were evaluated for coexpression of CD62L and CCR7 (B)(i) and Foxp3 expression (B) (ii). (C) Bulk CD4 cells and Tregs were restimulated for 24 h, and IC flow cytometry was performed to evaluate effector T cell coexpression of Foxp3 and IL-2 (i) or IFN-γ (ii). Results are mean ± SEM of n = 5 cultures. (D) Control CD4 cells and Tregs were compared for ability to suppress CD4+ (i) and CD8+ (ii) T cell proliferation over 5 d of culture in response to allogeneic DC (mean ± standard error of the mean [SEM] of n = 8 donors). (E) Representative data showing control CD4 and Treg suppression of responder CD4 (i) and CD8 (ii) proliferation over a range of suppressor cell to target ratios of 1∶20 to 1∶100. (0.22 MB TIF) [file pbio.1000302.s001.tif]

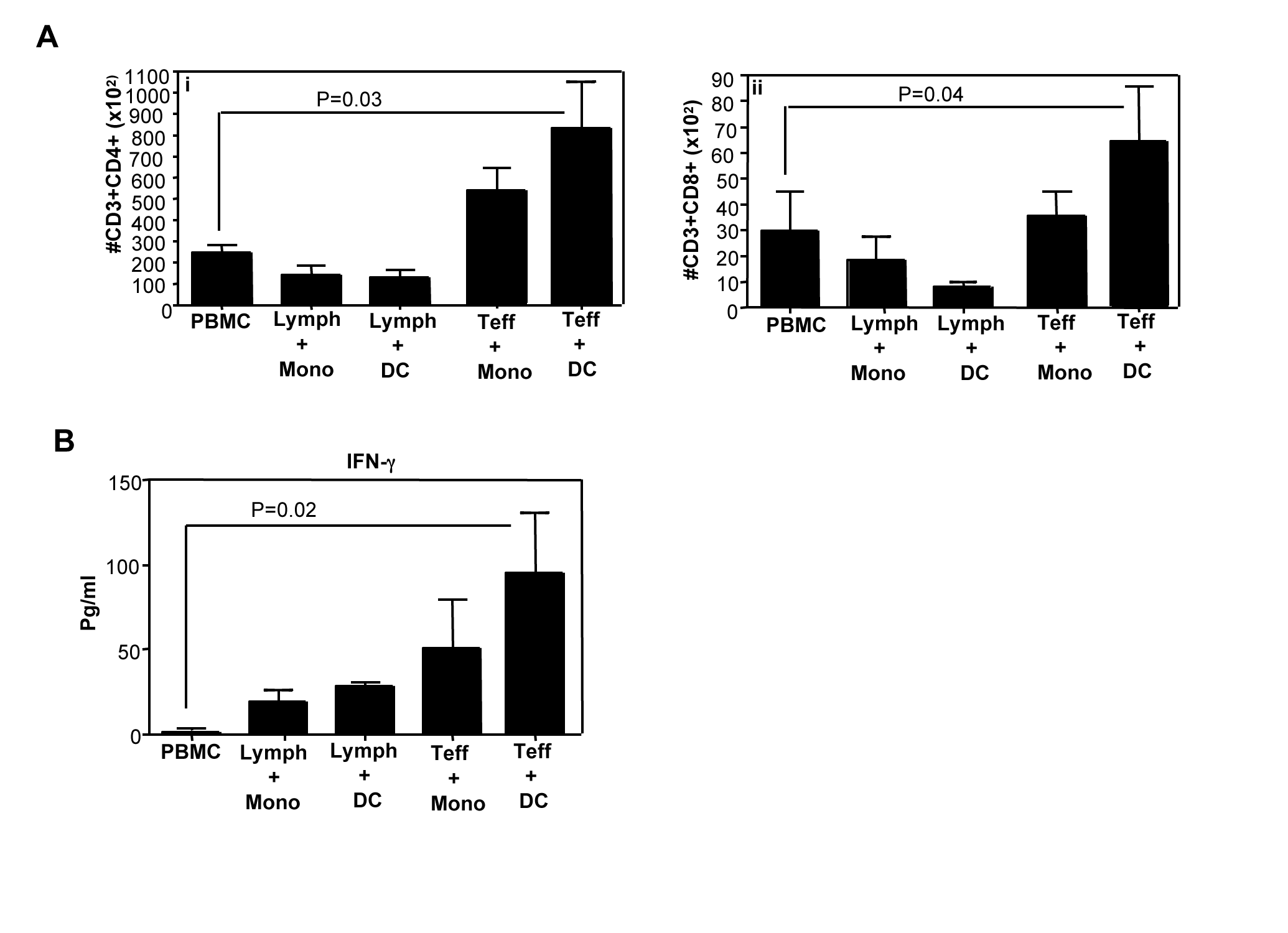

Supplement: Figure S2 — Human T cell numbers in vivo: role of ex vivo T cell activation. Rag2−/−γc−/− mice were reconstituted with human cells, as indicated, including: PBMC alone (“PBMC”), purified lymphocytes plus either monocytes (“Lymph+mono”) or DC (“Lymph+DC”), or ex vivo-expanded effector T cells alone plus either monocytes (“Teff+mono”) or DC (“Teff+DC”). The dose of effector T cells and APC populations were 1×10^7^ and 0.5×10^6^ cells per recipient, respectively. (A) On day 30 after transplant, the number of human CD4+ and CD8+ T cells in the spleen was determined using flow cytometry ((i) and (ii), respectively). (B) At day 30 posttransplant, splenic cells were costimulated using anti-human CD3 and CD28 beads; the resultant 24-h supernatant was then tested for content of IFN-γ by multiplex bead array. All results shown are the mean ± SEM of n = 10 recipients per cohort. An asterisk (*) indicates that the difference relative to the PBMC cohort was statistically significant (p<0.05). (0.14 MB TIF) [file pbio.1000302.s002.tif]
